# Comparative Assessment of Transcutaneous Bilirubin Versus Total Serum Bilirubin Measurements in Preterm Infants

**DOI:** 10.7759/cureus.97755

**Published:** 2025-11-25

**Authors:** Sirine Saleh, Manal Mouhssine, Satish C Nair, Usha Uthari, Mustafa Alabdullatif

**Affiliations:** 1 Pediatrics, Tawam Hospital, Al Ain, ARE; 2 Medical Research, PureHealth Group, Abu Dhabi, ARE; 3 Neonatology, Tawam Hospital, Al Ain, ARE

**Keywords:** bilirubin, general pediatrics, gulf cooperation council (gcc), jaundice, middle east and north africa (mena), neonatal care, preterm labour

## Abstract

Introduction: Hyperbilirubinemia is a common condition, particularly among preterm neonates. Although transcutaneous bilirubin (TCB) measurement offers a non-invasive modality for bilirubin assessment, its diagnostic accuracy in preterm populations remains incompletely characterized. This study addresses a knowledge gap by focusing on preterm neonates between 28 and 35 weeks of gestation and evaluating the concordance between TCB and total serum bilirubin (TsB) measurements.

Materials and methods: A prospective cohort study was conducted at a tertiary care academic medical center between April 2023 and February 2024. The cohort included preterm neonates (≤35 weeks of gestation) admitted to the Neonatal Intensive Care Unit (NICU). TCB measurements were obtained within two hours of each TSB sampling using BiliCare™, a transcutaneous bilirubin meter. Statistical analyses, including Bland-Altman plots and Lin's concordance correlation coefficient (CCC), were used to evaluate the agreement between the two methods, TCB and TsB.

Results: A total of 300 paired bilirubin measurements from preterm neonates (gestational age range: 24-35 weeks) were analyzed. TCB and TsB demonstrated substantial concordance, with CCC values of 0.88 for neonates born at ≤28 weeks, 0.92 for those born at 29-32 weeks, and 0.95 for those born at>32 weeks. Minor variability and systematic bias were noted. The limits of agreement across all groups fell within a clinically acceptable range of ±4 mg/dL.

Conclusion: TCB represents a viable non-invasive alternative to TsB for bilirubin monitoring in preterm neonates with hyperbilirubinemia, potentially minimizing the need for frequent invasive blood sampling. However, the observed variability across gestational age subgroups underlines the need for cautious interpretation, particularly in subpopulations where discrepancies may occur. Further investigations are warranted to optimize the application of TCB in larger neonatal cohorts.

## Introduction

Elevated levels of bilirubin characterize neonatal hyperbilirubinemia in a newborn's blood, which causes a yellow discoloration of the skin and eyes, resulting from an imbalance between bilirubin production and excretion. If severe, it can lead to serious complications. Approximately 60% of term neonates and 80% of preterm neonates develop jaundice within the first week of life, making accurate and timely bilirubin assessment a cornerstone of neonatal care [1]. Left untreated, severe hyperbilirubinemia can lead to devastating neurological sequelae, including kernicterus, underscoring the need for precise monitoring and management strategies [2].

Preterm infants are at an elevated risk of hyperbilirubinemia due to immature liver function, increased hemolysis, and greater permeability of the blood-brain barrier. These factors contribute to the heightened vulnerability of this population to significant or dangerous levels of hyperbilirubinemia and its long-term sequelae [2]. Globally, the implementation of universal bilirubin screening has significantly reduced the incidence of extreme hyperbilirubinemia (defined as total serum bilirubin (TSB) ≥30 mg/dL) [3]. However, challenges in accurately assessing bilirubin levels in preterm neonates persist.

In clinical practice, TSB is the gold standard for bilirubin measurement, but its invasive nature, technical requirements, and associated discomfort necessitate alternative approaches. Transcutaneous bilirubin (TCB) measurement offers a non-invasive and rapid method for bilirubin screening; however, its accuracy and reliability in preterm neonates, particularly those born at earlier gestational ages, remain incompletely characterized. While studies have validated TCB use in term neonates [4,5], the literature evaluating its accuracy in preterm populations is limited and inconsistent. Certain studies report a strong correlation between TCB and TSB in preterm neonates, while others highlight discrepancies, particularly in extremely preterm infants [6-8]. This variability stresses the need for further research to refine TCB application in this vulnerable group.

The selection of this gestational age range reflects clinical observations suggesting that bilirubin metabolism and TCB measurement reliability differ significantly in neonates born before 35 weeks compared to those closer to term. Specifically, this study aims to assess the accuracy and reliability of TCB compared to TSB in preterm neonates admitted to Tawam Hospital. We hypothesize that TCB measurements provide a sufficiently accurate, non-invasive alternative to TSB for monitoring bilirubin levels in preterm neonates. By evaluating the agreement between TCB and TSB, this study seeks to determine the potential for TCB to reduce the frequency of invasive blood sampling, improving clinical efficiency while maintaining diagnostic reliability.

## Materials and methods

A prospective cohort study was conducted at Tawam Hospital, Al Ain, UAE, between April 2023 and February 2024. These 10 months were chosen to ensure an adequate sample size while minimizing the risk of seasonal biases and providing sufficient data for a robust analysis of the reliability of TCB measurements in preterm infants requiring extended care and monitoring in the Neonatal Intensive Care Unit (NICU).

Care protocols and guidelines

Bilirubin assessment, phototherapy initiation, and sampling were followed as per established hospital protocols and international guidelines. Infants received standard supportive care during phototherapy, including hydration and skin protection.

Study population and inclusion criteria

Preterm infants born before or at 35 weeks of gestation were included, as they are at higher risk of bilirubin-related complications than near-term or term neonates [9]. Although prematurity is defined as birth before 37 weeks of gestation, the cutoff of 35 weeks was selected because TCB is reliable in neonates beyond this gestation. Additionally, neonates born between 35 and 37 weeks often exhibit bilirubin metabolism more similar to that of term infants. This study specifically addresses the knowledge gap in evaluating TCB accuracy in neonates with a gestational age of ≤35 weeks. Congenital anomalies (e.g., Down syndrome, hydrops fetalis) and diffuse skin conditions (e.g., severe bruising or skin infections) were excluded to minimize confounding variables that could interfere with TCB accuracy.

TCB measurement procedure

TCB was measured using the BiliCare™ Transcutaneous Bilirubin Meter (Mennen Medical), calibrated weekly per manufacturer recommendations [10]. Although no validation study has been conducted in preterm populations, the device's accuracy and reliability have been demonstrated in term neonates [10]. The device uses spectrophotometric technology to measure bilirubin levels through soft tissue. Measurements were taken from the ear pinna, chosen for its high perfusion, accessibility, and because the device manufacturer recommended it. A single TCB measurement was obtained at the time of each TSB test. Multiple measurements were not taken from the same site to minimize discomfort and variability. Nurses and doctors were trained on the proper use of the device to reduce operator-related inconsistencies. During phototherapy and, for 24 hours afterwards, TCB measurements were avoided, based on evidence showing poor correlation between TCB and TSB levels during this period. TSB measurements remained the standard for bilirubin monitoring under such conditions. TCB measurements were excluded during phototherapy and for 24 hours following phototherapy due to poor correlation with TSB. Manufacturer recommendations and previous studies guided this exclusion.

TSB measurement procedure

The majority of TSB samples were collected via capillary sampling, depending on clinical indication. Samples were transported in opaque covers to prevent light-induced bilirubin degradation. Analysis following stratification was performed promptly using the Roche/Hitachi Cobas C systems and the colorimetric diazo method to minimize delays and degradation.

Study design and statistical analysis

This study was designed to evaluate the agreement between TCB and TSB measurements. Multiple TCB and TSB measurements were collected per infant over the study period. Data were analyzed using Lin's concordance correlation coefficient (CCC) [11], which assesses both precision and accuracy. Bland-Altman plots enabled the visualization of the agreement between methods and identified systematic biases [12]. Figures have been combined for clarity, with axes labeled appropriately and legends simplified for easier interpretation. Since each neonate contributed multiple measurements, statistical models accounted for repeated measures to avoid overestimating agreement. A mixed-effects model was used to account for within-subject variability.

Sample size calculation

The sample size calculation was performed manually using the standard single-proportion formula:

n=(Z^2×p×(1-p))/e^2 ÷(1+(Z^2×p×(1-p))/(e^2×N))

where Z = 1.96 (corresponding to a 95% confidence level), p = 0.60 (estimated proportion of altered bilirubin), e = 0.05 (margin of error), and N = 500 (annual NICU admissions). The calculated minimum required sample size was 214 neonates. The study used a convenience sampling method of all preterm neonates admitted to the NICU during the study period [13].

Ethical considerations

The study was approved by the Institutional Ethics Committee (MF2058-2023-925) at Tawam Hospital. Parental consent was obtained for participation, and data were anonymized to ensure patient confidentiality.

## Results

During the study period, 650 neonates were admitted to the NICU. Among them, 320 were preterm (42%, 320/650). A total of 214 (67%, 214/320) preterm neonates met the inclusion criteria and were enrolled in the study. A total of 300 paired TCB-TSB measurements were obtained from enrolled neonates, with each infant contributing an average of 1.4 paired measurements (not a measure of variability). Out of the 214 enrolled neonates, the majority were male (Table 1) and delivered via cesarean section. UAE nationals were the predominant maternal nationality (Table 1).

**Table 1:** The gender, mode of delivery, and maternal nationality are shown for the infants

Category	Subcategory	Frequency (%)
Gender	Male	126 (59)
	Female	88 (41)
Mode of Delivery	Cesarean section	171 (80)
	Normal vaginal delivery	43 (20)
Maternal Ethnicity	Emirati (Nationals)	137 (64)
	Non-Emirati (Expatriates)	77 (36)

Table 2 indicates that the median gestational age of the enrolled neonates was 33 weeks (interquartile range (IQR): 30-34), the mean birth weight was 1.7 kg (SD: 0.5), with a range of 0.45-2.9 kg, and the median age at which samples were collected was 72 hours (IQR: 12-404).

**Table 2:** The gestational weeks of the infants in the study birth weight and ages are shown, represented as minimum, mean, median, and maximum

Variables	Minimum	Mean	Median	Maximum
Gestational age (weeks)	24	31	33	35
Birth weight (kilograms)	0.45	1.7	1.8	2.9
Age of newborns where samples collected (hours)	12	95	72	404

**Figure 1:**
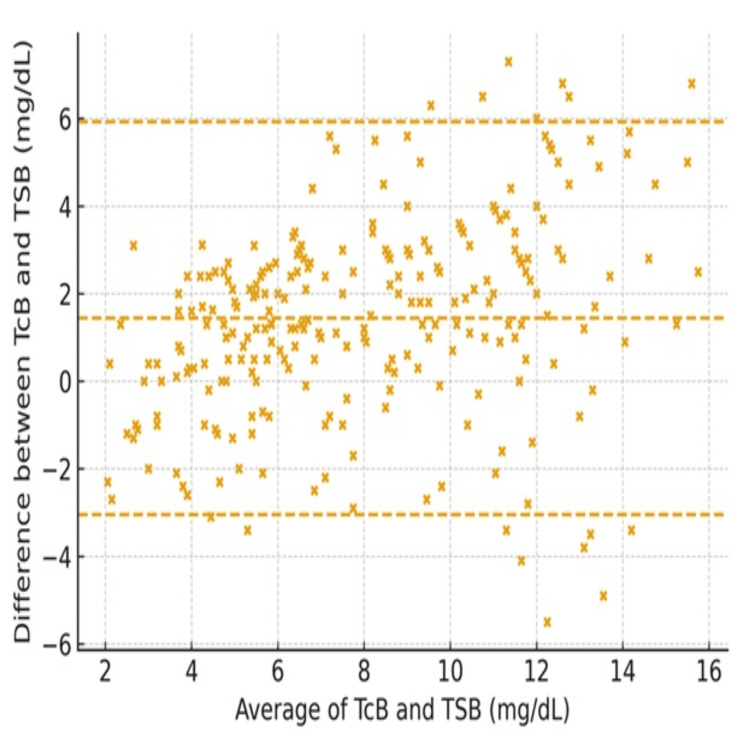
Bland–Altman plot for all patients (axes in mg/dL) Transcutaneous bilirubin (TCB) and total serum bilirubin (TSB) in milligrams per deciliter. The Bland–Altman plot shows agreement between TcB and TSB measurements. The dashed lines represent the mean bias (middle) and 95% limits of agreement (upper and lower). Mean bias = +1.5 mg/dL (95% CI: +1.2 to +1.8); 95% LOA = −3.2 to +6.2 mg/dL.

The average of TCB and TSB measurements for all patients showed substantial agreement, as indicated by the concordance correlation coefficient (Figure 1). The Bland-Altman analysis showed a mean bias of +1.5 mg/dL (95% CI: +1.2 to +1.8), with 95% limits of agreement ranging from -3.2 to +6.2 mg/dL (95% CI for LOA: lower -3.8 to -2.6; upper +5.6 to +6.8). This indicates that TcB readings were on average 1.5 mg/dL higher than corresponding TSB values, with acceptable agreement across the measurement range. Despite the small-scale and location shift, suggesting slight differences in variability and systematic bias, the bias correction factor remained close to 1, indicating that the two methods are largely comparable with minimal systematic bias (Figure 1).

**Figure 2:**
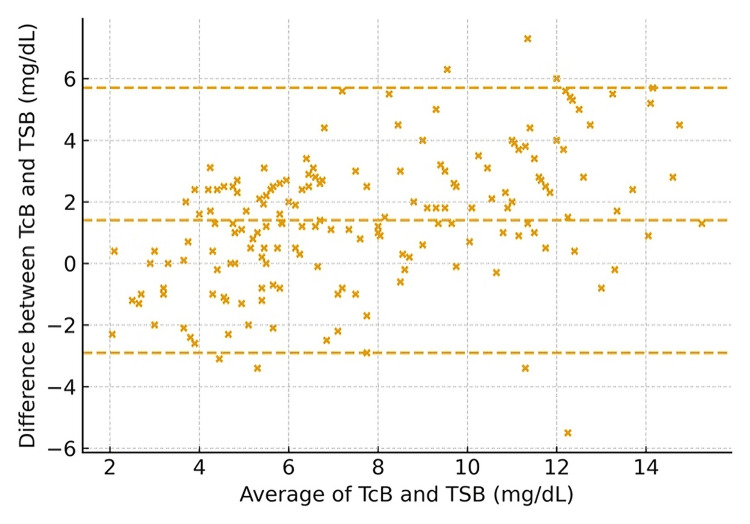
Bland–Altman plot by maternal ethnicity, UAE nationals (Emirati) (axes in mg/dL) Transcutaneous bilirubin (TCB) and total serum bilirubin (TSB) in milligrams per deciliter

**Figure 3:**
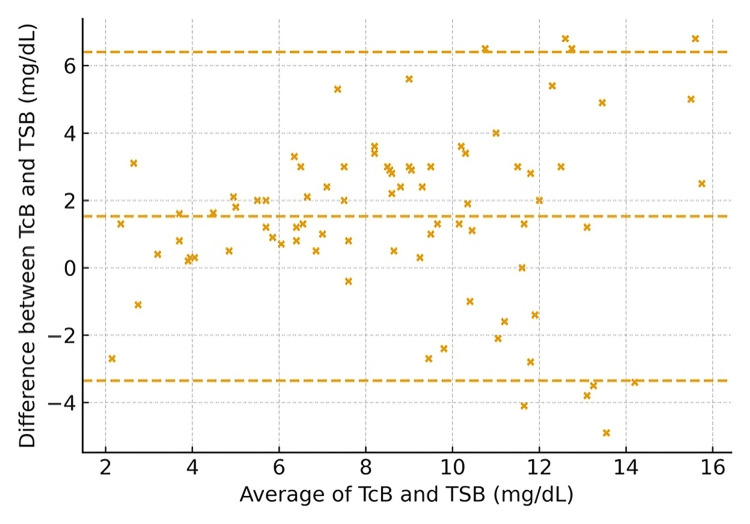
Bland–Altman plot by maternal ethnicity, non-nationals (expatriates) (axes in mg/dL) Transcutaneous bilirubin (TCB) and total serum bilirubin (TSB) in milligrams per deciliter

**Figure 4:**
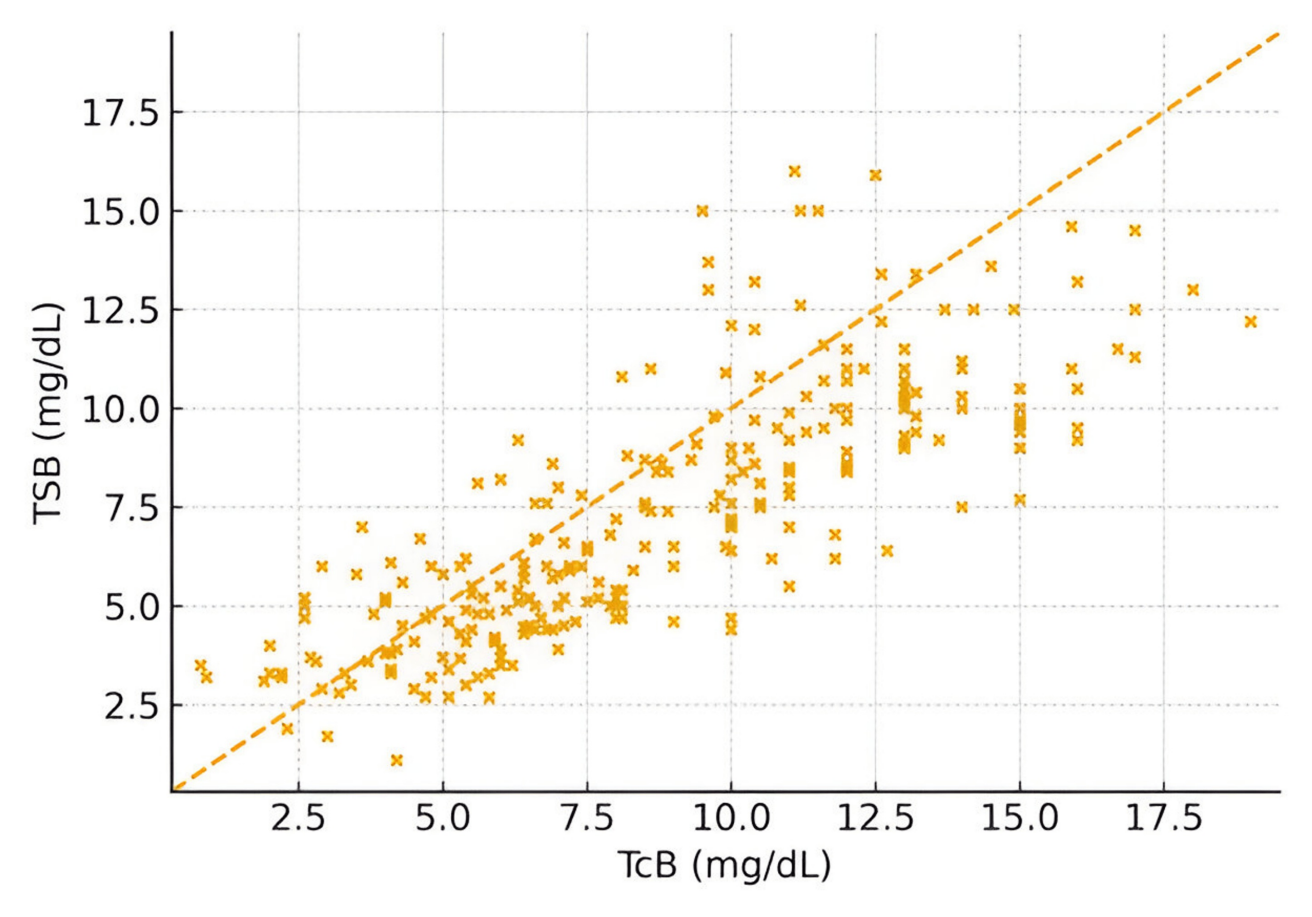
Scatter plot of TcB vs. TSB with line of identity (axes in mg/dL) Transcutaneous bilirubin (TCB) versus total serum bilirubin (TSB) in milligrams per deciliter

Furthermore, measurement agreement was demonstrated using maternal ethnicity for UAE nationals (Figure 2) and non-nationals (Figure 3). The scatter plot indicates a generally strong positive correlation between TCB and TSB, suggesting that TCB can be used as a substitute for TSB. However, the scatter around the line of identity and the presence of outliers indicate variability (Figure 4).

**Figure 5:**
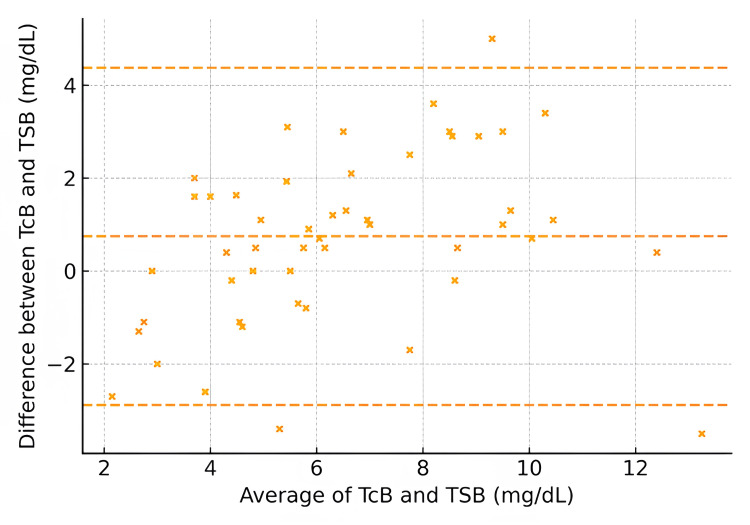
Bland–Altman plot by gestational age: ≤28 weeks (axes in mg/dL) Transcutaneous bilirubin (TCB) and total serum bilirubin (TSB) in milligrams per deciliter

**Figure 6:**
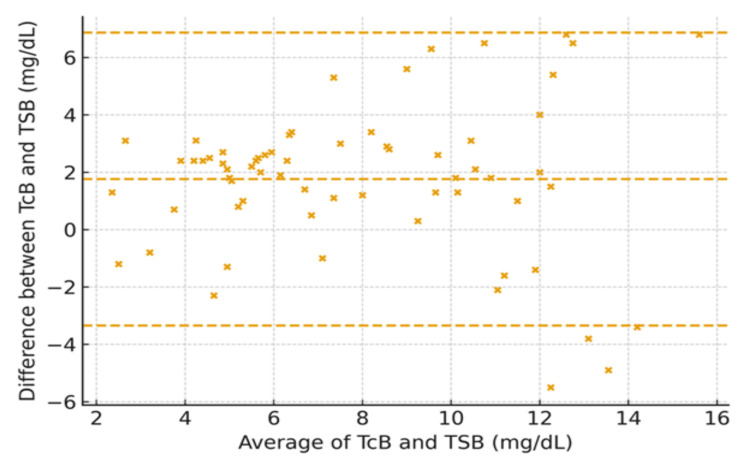
Bland–Altman plot by gestational age: 28–32 weeks (axes in mg/dL) Transcutaneous bilirubin (TCB) and total serum bilirubin (TSB) in milligrams per deciliter

**Figure 7:**
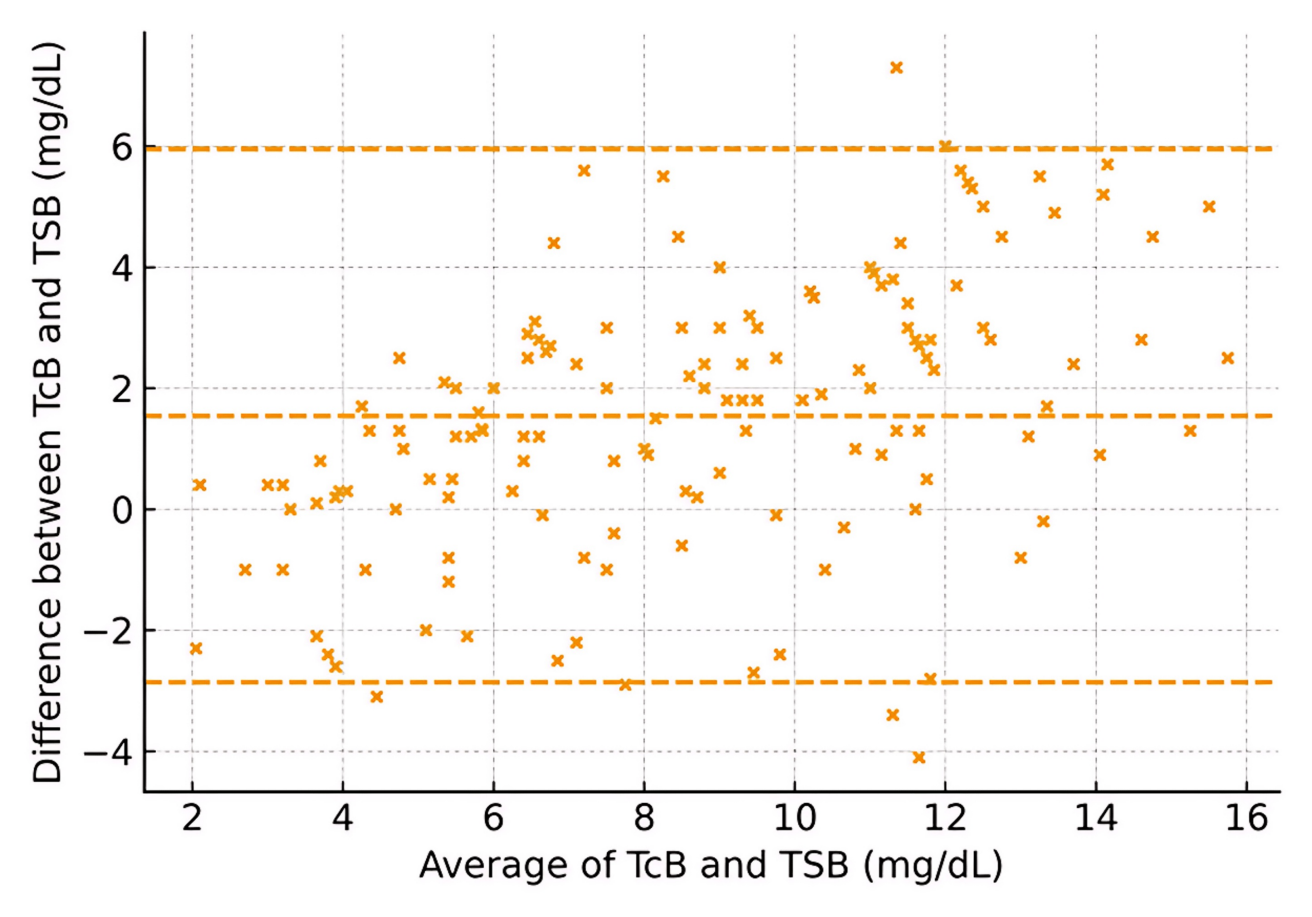
Bland–Altman plot by gestational age: ≥32 weeks (axes in mg/dL) Transcutaneous bilirubin (TCB) and total serum bilirubin (TSB) in milligrams per deciliter

It is noteworthy that for neonates born ≤28 weeks, the CCC was 0.88, reflecting moderate agreement with a bias of +1.2 mg/dL. Neonates born between 29 and 32 weeks had a CCC of 0.92, with a low bias of +0.8 mg/dL. Those born >32 weeks demonstrated the highest agreement, with a CCC of 0.95 and minimal bias (+0.5 mg/dL) (Figures 5-7).

**Figure 8:**
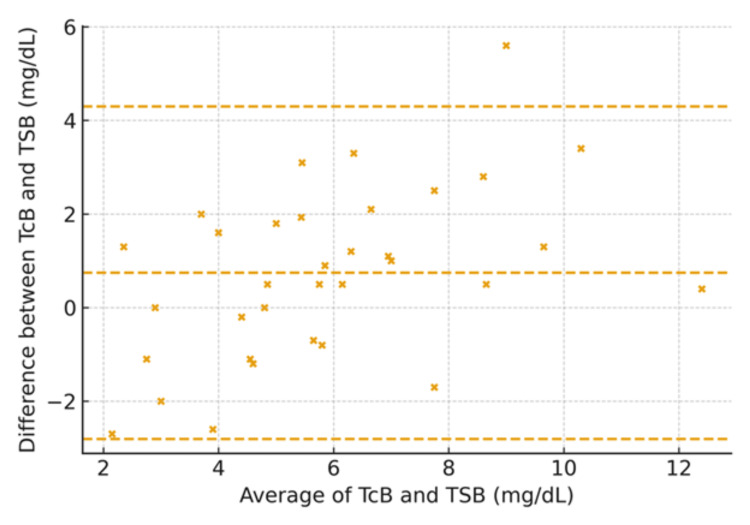
Bland–Altman plot by birth weight: <1 kg (axes in mg/dL) Transcutaneous bilirubin (TCB) and total serum bilirubin (TSB) in milligrams per deciliter

**Figure 9:**
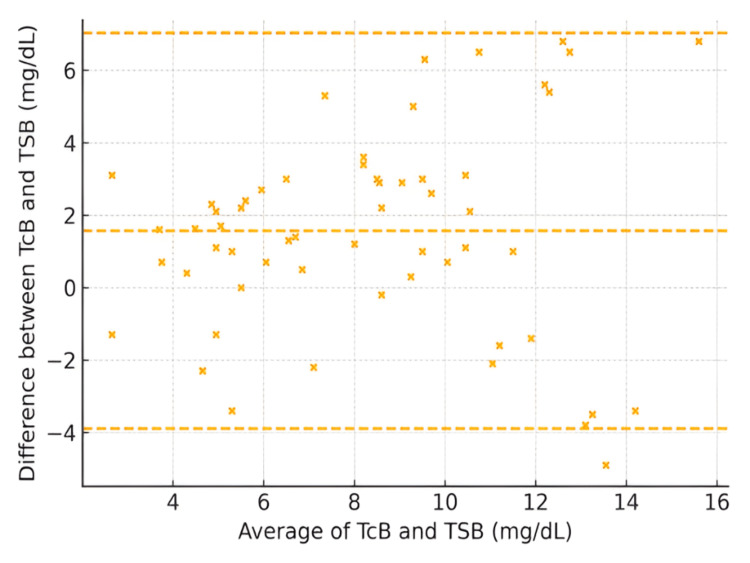
Bland–Altman plot by birth weight: 1–1.5 kg (axes in mg/dL) Transcutaneous bilirubin (TCB) and total serum bilirubin (TSB) in milligrams per deciliter

Nevertheless, the Bland-Altman analysis by weight category revealed greater variability in neonates <1 kg and >1.5 kg. The CCC values were 0.87 and 0.91, for neonates <1 kg, and 1-1.5 kg, respectively (Figures 8, 9). A strong positive correlation was observed between TcB and TSB values (r = 0.86, 95% CI: 0.82-0.89, p < 0.001). Lin’s concordance correlation coefficient also demonstrated good agreement between the two measurement methods (CCC = 0.83, 95% CI: 0.79-0.86). These findings, together with the Bland-Altman analysis (mean bias = +1.5 mg/dL; 95% LOA = -3.2 to +6.2 mg/dL), confirm that TcB provides a reliable noninvasive estimate of serum bilirubin within clinically acceptable limits. Additionally, neonates not undergoing phototherapy, TCB demonstrated a CCC of 0.93 (95% CI: 0.91-0.95) (data not shown).

## Discussion

This study evaluated the agreement between transcutaneous bilirubin and total serum bilirubin measurements in preterm neonates born at ≤35 weeks' gestation, a critical subgroup with heightened vulnerability to bilirubin-related complications. Our results highlight subgroup-specific variability in TCB accuracy, contributing valuable insights to an area where the literature remains inconsistent [14]. Earlier reports have established that TCB is a reliable screening tool for hyperbilirubinemia. However, treatment decisions must rely on TSB given the limitations of TCB accuracy at extreme bilirubin levels [1]. Nevertheless, it also noted that significant variability across gestational age and weight subgroups, with the greatest discrepancies observed in extremely preterm neonates (<28 weeks) and low-birthweight infants (<1 kg) [15]. In our study, the limits of agreement (LOA) between TCB and TSB were within ±4 mg/dL for 95% of measurements, indicating acceptable clinical utility [14].

The observed variability aligns with earlier findings that TCB accuracy is influenced by factors such as skin pigmentation, gestational age, and bilirubin level [2-4]. Slusher et al. [5] noted similar challenges in diverse populations, where TCB results varied significantly with melanin levels, underscoring the need for population-specific validation [5]. Additionally, reports have highlighted a reduced TCB reliability at higher bilirubin levels, which might explain the discrepancies observed in our subgroup analyses [6,7]. The Bland-Altman analysis for gestational age subgroups in our study revealed distinct patterns of agreement between the two measurement methods across different gestational age categories. For infants born at or before 28 weeks of gestation, the plot indicated a consistent bias, with differences between methods showing little variation across the range of averages, suggesting a systematic discrepancy. In the 28-32 weeks gestational age group, agreement between methods was improved, with a narrower spread of differences, although some bias persisted. In contrast, for infants born after 32 weeks of gestation, the plot demonstrated the greatest variability in agreement, with a wider range of differences and less consistent bias. These findings suggest that, while the measurement methods are generally comparable, the degree of agreement varies significantly with gestational age, particularly in the most preterm and near-term groups, where systematic differences are more pronounced. Our study extends further by focusing specifically on neonates at ≤35 weeks' gestation and analyzing subgroup-specific data. For example, TCB demonstrated lower agreement in neonates ≤28 weeks of gestation (CCC = 0.88) and improved concordance in those born >32 weeks (CCC = 0.95). These findings reinforce the need for cautious interpretation of TCB results in extremely preterm neonates, where systemic biases may arise due to the immature properties of the skin and bilirubin metabolism [8]. Additionally, our study also highlights the importance of device calibration and adherence to manufacturer guidelines. The variability in TCB accuracy across devices underscores the need for clinicians to understand the limitations of their equipment and adapt clinical protocols accordingly [16]. While TCB offers significant advantages in reducing invasive procedures, these findings suggest that its use should be complemented by TSB, particularly in neonates at the extremes of prematurity or with high bilirubin levels.

This limitation of the study in accurately measuring bilirubin levels between the two methods may be affected by the capillary and venous samples, which may introduce minor variability, despite standardized procedures. Additionally, stratification by delivery mode was ensured to limit potential physiological differences, and further studies are needed to confirm these findings. Other contributing factors, such as phototherapy, blood type, and critical care interventions, were not analyzed, also important for the broader validation of bilirubin measurements in preterm populations. Interestingly, while TCB has been extensively validated in term-neonates, its performance in preterm populations may require more robust, multicenter validation.

## Conclusions

In conclusion, despite limitations for the accurate bilirubin estimation in preterm neonates, TCB remains a valuable, non-invasive tool for hyperbilirubinemia screening. However, TCB cannot replace TSB for treatment decisions. Clinicians should take into consideration subgroup-specific variability and exercise caution when interpreting TCB results in extremely preterm and low-birthweight neonates. Further research is warranted to identify and mitigate factors affecting TCB accuracy, thus enabling its broader and safer application in neonatal care.
